# Non-invasive scoring of cellular atypia in keratinocyte cancers in 3D LC-OCT images using Deep Learning

**DOI:** 10.1038/s41598-021-04395-1

**Published:** 2022-01-10

**Authors:** Sébastien Fischman, Javiera Pérez-Anker, Linda Tognetti, Angelo Di Naro, Mariano Suppa, Elisa Cinotti, Théo Viel, Jilliana Monnier, Pietro Rubegni, Véronique del Marmol, Josep Malvehy, Susana Puig, Arnaud Dubois, Jean-Luc Perrot

**Affiliations:** 1 DAMAE Medical, Paris, France; 2 Melanoma Unit, Hospital Clinic Barcelona, University of Barcelona, Barcelona, Spain; 3 CIBER de enfermedades raras, Instituto de Salud Carlos III, Barcelona, Spain; 4 Dermatology Unit - Department of Medical, Surgical and Neurological Sciences, University of Siena, Siena, Italy; 5 Department of Dermatology, Université Libre de Bruxelles, Hôpital Erasme, Brussels, Belgium; 6 Groupe d’Imagerie Cutanée Non Invasive (GICNI) of the Société Française de Dermatologie (SFD), Paris, France; 7 Institut Jules Bordet, Université Libre de Bruxelles, Brussels, Belgium; 8 Department of Dermatology and skin cancer, la Timone hospital, Assistance Publique-Hôpitaux de Marseille, Aix-Marseille University, Marseille, France; 9 Université Paris-Saclay, Institut d’Optique Graduate School, Laboratoire Charles Fabry, Palaiseau, France; 10 Department of Dermatology, University Hospital of Saint-Etienne, Saint-Etienne, France

**Keywords:** Diagnostic markers, Applied optics, Skin cancer, Computer science

## Abstract

Diagnosis based on histopathology for skin cancer detection is today’s gold standard and relies on the presence or absence of biomarkers and cellular atypia. However it suffers drawbacks: it requires a strong expertise and is time-consuming. Moreover the notion of atypia or dysplasia of the visible cells used for diagnosis is very subjective, with poor inter-rater agreement reported in the literature. Lastly, histology requires a biopsy which is an invasive procedure and only captures a small sample of the lesion, which is insufficient in the context of large fields of cancerization. Here we demonstrate that the notion of cellular atypia can be objectively defined and quantified with a non-invasive in-vivo approach in three dimensions (3D). A Deep Learning (DL) algorithm is trained to segment keratinocyte (KC) nuclei from Line-field Confocal Optical Coherence Tomography (LC-OCT) 3D images. Based on these segmentations, a series of quantitative, reproducible and biologically relevant metrics is derived to describe KC nuclei individually. We show that, using those metrics, simple and more complex definitions of atypia can be derived to discriminate between healthy and pathological skins, achieving Area Under the ROC Curve (AUC) scores superior than 0.965, largely outperforming medical experts on the same task with an AUC of 0.766. All together, our approach and findings open the door to a precise quantitative monitoring of skin lesions and treatments, offering a promising non-invasive tool for clinical studies to demonstrate the effects of a treatment and for clinicians to assess the severity of a lesion and follow the evolution of pre-cancerous lesions over time.

## Introduction

Histopathology is the gold standard to confirm a diagnosis in all tissues. The advent of numerical technologies facilitate the access of physicists to digital imaging. However, diagnoses and prognoses with whole slide images still suffer from the subjectivity and level of experience of the specialist, even when some grading scales systems exist^1,2^.

The recent progress in computer vision with Deep Learning techniques has opened up new opportunities to create more reliable quantitative metrics based on physical segmentations to help pathologists. Specifically, metrics based on cell nuclei spatial distributions have gained recent interest as cell nuclei are essential markers for the diagnosis and study of cancer^3^. Due to the large amount of cells visible in medical images at microscopic level, their global geometry is especially hard to understand for the human eye, making automated segmentation of nuclei particularly helpful and promising. Waliszewski et al.^4^ tried to quantify the spatial distribution of cancer cell nuclei with a fractal geometrical model to automate Gleason scoring. Kendall et al.^5^ introduced geoscience frameworks and a two dimensional space (2D) graph-based approach for digital pathology in order to quantitatively differentiate lesional and non-lesional images. Lu et al.^6^ also used automated cell nuclei segmentations and a local nuclear graph approach to create complex, hardly interpretable but reproducible metrics, useful for predicting lung cancer survival of patients. Jiao et al.^7^ proposed a 2D distribution analysis of spatial organisation of cell nuclei in brain tumors using Voronoi statistics. Zhou et al.^8^ applied a graph neural network approach from nuclei segmentation in order to automatically grade colorectal cancer histology images.

All these studies tried to capture the complexity of the spatial distribution of cell nuclei from histology slides into a few quantitative metrics to demonstrate predictive or discriminative power. But histology slides cannot perfectly reflect the actual physical changes of tissues. In fact, slide preparation pipeline (including biopsy, tissue fixation, processing, sectioning and staining) creates physical deformation and results in a 2D representation that cannot fully capture the spatial complexity of a 3D reality, which is a known issue^9–11^.

LC-OCT is a new in-vivo non-invasive medical imaging technology that combines deep penetration and cellular resolution in 3D^12^. It allows to study cell nuclei distributions without the sliding procedure deformation and renders information in 3D. With a resolution of 1 µm, LC-OCT is more accurate than standard Optical Coherence Tomography (OCT)^13^ and presents an ideal resolution for nuclei segmentation. Reflectance Confocal Microscopy (RCM) images are very similar to LC-OCT images, except that they don’t allow imaging in 3D. Pellacani et al.^14^ showed that two by two comparisons of 2D RCM images could allow specialists to rank actinic keratosis by atypia in a similar order than with histopathology images. Such an approach does not allow to objectively define atypia nor systematically reproduce the results, and no absolute atypia score is generated, only a relative score among a fixed set of images.

This study proposes a novel automated approach based on deep learning segmentation applied to 3D LC-OCT images, capable of accurately assessing the amount of atypia in keratinocyte cancers. Our approach overcomes many of the limitations of existing studies. Our pipeline is non-invasive and therefore does not require biopsies. Atypia scores are calculated on 3D LC-OCT images and therefore do not suffer from the distortions and limitations of a 2D histology slide which is also a time consuming process whereas a 3D LC- OCT image can be acquired in 30 seconds. All features used to define cellular atypia are physically robust and easy to interpret, making the final score more reliable than that provided by ”black box” algorithms.

## Material

### Line-field confocal optical coherence tomography (LC-OCT)

**Figure 1:**
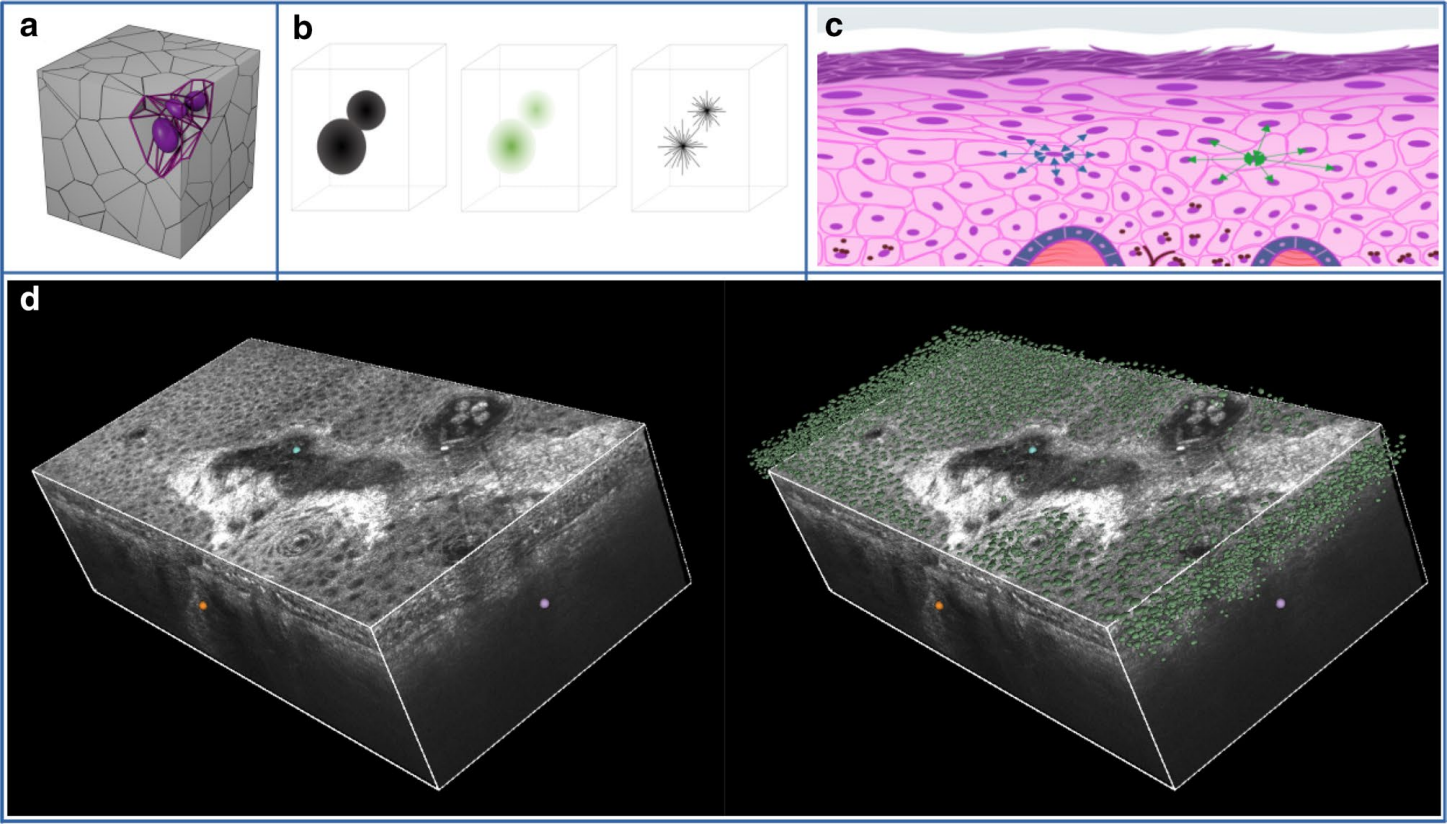
(**a**) Representation of a 3D Voronoi Diagram in a cube. (**b**) Scheme of a StarDist inputs, probability predictions and rays predictions. (**c**) Representation of skin structure, center to center distance (in green) and border to border distance (in blue). (**d**) Example of 3D visualisation of StarDist nuclei detection in LC-OCT 3D images.

Images of the study were collected using LC–OCT devices (DAMAE Medical, Paris) which produce—painlessly and non-invasively— vertically-oriented (histology-like) and horizontally-oriented (similar to Reflectance Confocal Microscopy (RCM)) sectional images as well as full 3D volume block images (Fig. 1). The LC-OCT technology uses a two-beam interference microscope with a supercontinuum laser as a class 1 light source at the center wavelength of 800 nm and a line-scan camera as a detector. LC-OCT measures the time of flight and amplitude of light backscattered from the tissue microstructures illuminated by a line of light. This new technology combines the OCT interferometry principle with the confocal spatial filtering of RCM. The vertical and horizontal sectional images are produced in real time at 8 frames per second. 3D stacks can be acquired for 3D reconstruction in approximately 30 s per stack. The images can be acquired up to a depth of $\sim$ 500 µm. They have an axial resolution of 1.1 µm, a lateral resolution of 1.3 µm and a field of view of 1.2 mm × 0.4 mm (vertical) and 1.2 mm × 0.5 mm (horizontal). Complete technical details are described elsewhere^15–17^.

### Patients and volunteers

In order to compare healthy skin with pathological skin we retrospectively analyzed a total of 185 LC-OCT 3D images (Table 1). For lesional skin, we retrospectively collected consecutive cases of 35 patients from 4 hospitals—Hôpital Erasme (Belgium), Hospital Clinic Barcelona (Spain), University Hospital of Saint-Etienne (France), University of Siena (Italy)—imaged with LC-OCT 3D between January 2020 and May 2021 with an histopathologically confirmed diagnosis of Actinic Keratosis (AK), subclinical AK (SAK)—area surrounding the AK lesion within the field of cancerization—or Bowen disease and for which image quality was judged appropriate regarding KC visibility (entire epidermis visible without too thick hyperkeratosis). Healthy skin images have been acquired between February 2020 and January 2021 on healthy volunteers in Paris for DAMAE Medical internal research. All patients and volunteers gave informed consent for their images to be anonymously used in this study.

From a clinical standpoint Bowen’s disease (Bowen) is the most severe pathology, followed by Actinic Keratosis (AK) and then the subclinical AK. Even though Bowen and AK are different diseases they present similarities and may represent the same disease process at different stages^18^.

**Table 1:** Demographics of the healthy and pathological populations.

	Healthy	Pathological	Subclinical AK	AK	Bowen
Number of patients	38	34	18	22	7
Number of woman (percentage)	38 (100%)	10 (29%)	2 (11%)	6 (27%)	2 (28%)
Number of lesions	114	71	34	30	7
Age (average ± standard deviation)	49.6±10.0	75.6±9.8	77.5±7.6	74.7±11.0	66±13.3
**Positions**					
Head/neck	90 (78%)	62 (88%)	34 (100%)	26 (87%)	2 (29%)
Trunk	12 (11%)	4 (6%)	0 (0%)	2 (7%)	2 (29%)
Upper extremities	12 (11%)	0 (0%)	0 (0%)	0 (0%)	0 (0%)
Lower extremities	0 (0%)	4 (6%)	0 (0%)	1 (3%)	3 (42%)

### 3D keratinocytes nuclei segmentation with Deep Learning

In order to detect cells and segment them individually, we used an instance segmentation approach. We trained a 3DStarDist^19^ model, a deep learning architecture well suited for this problem as it learns directly on 3D images thanks to 3D convolutions and detects star-convex polyhedras similar to cell nuclei.

Each 3D LC-OCT image was segmented using our trained model as visualized in Fig. 1. In the combined images of this study, a total of more than 3.7 million nuclei were detected by the Deep Learning model with 2.5 millions nuclei from healthy images, 491000 in AK, 630000 in fields of cancerization and 95000 in Bowens. The predictions of the model have been reviewed and validated by an expert in LC-OCT image interpretation to ensure both good image quality and correct segmentation. More information about the training procedure and parameters can be found in the “Methods” section.

### Physical and biological metrics based on 3D nuclei segmentation

Based on keratinocyte nuclei segmentations we can compute different physical, quantitative and reproducible metrics in order to analyse the differences between healthy and pathological skins. Being able to easily interpret those metrics is important for building trust in the final results for practitioners, as explainable Artificial Intelligence is now largely preferred to black-box models when it comes to anatomic pathology^20^.

#### Graph based approach

Even though only cell nuclei are segmented from the LC-OCT images, the entire epidermis is actually filled with cells which closely adjoin each other^21^. A common modelization of this biological fact is done using Voronoi graphs. A Voronoi diagram^22,23^ partitions the space into 3D regions (also called Voronoi cells) where each nucleus center is the closest center to its own region (see Fig. 1 for illustration). The Voronoi graph is created using Delaunay triangulation^24^. Nodes are defined as the centers of the nuclei, and edges connect each cell to its neighbours closer than 50 µm.

#### Cell level metrics

From the raw segmentations and the Voronoi graph, we created 13 cell level features described in Table 2. These biologically and physically relevant, easy to interpret features are taking advantage of the 3D structures of the segmentations. They allow a more complex and trustful representation than previous similar work^7,25^. Furthermore, they are entirely derived from the 3D geometry of the detection of StarDist and do not use direct information from the original 3D image.

**Table 2:** Cell level metrics, list and descriptions.

Metric (feature name)	Description	Unit
Nucleus volume (volume)	Volume of the star-convex polyhedra detected by the StarDist model.	$mm^3$
Nucleus compactness (compactness)	A score that captures how close to a perfect sphere the detected nucleus is. It computes the area of a perfect sphere with the same volume as the detected nuclei and divides it by the actual area of the polyhedra: $\text {compactness}=\dfrac{(36*\pi *V^2)^{1/3}}{A}$ A value close to 1 indicates that the nucleus is very close to a sphere, while a lower value indicates a nucleus with a flatter shape.	None
Volume over compactness ratio (volume_over_compactness)	Ratio between volume and compactness.	$mm^3$
Number of neighbours (nb_neighbours)	Inside the Voronoi graph, this is the number of edges for the node. This is the number of adjacent neighbours for the considered cell.	None
Average center to center distance from neighbours (neighbor_dist)	Average distance to cell neighbours, from center to center. This is also a proxy for the entire cell diameter, as the distance between two adjacent cells is the sum of both radiuses, and we perform over multiple neighbours in all directions. (see Fig. 1).	$\mu m$
Average border distance to neighbours (border_dist)	Average distance to neighbours, from border of the nucleus to border of the neighbour nucleus. This is a proxy for the cytoplasm thickness(see Fig. 1).	$\mu m$
Average border distance over center distance ratio (border_over_neigh_distances)	Accounts for the ratio of the volume taken by the nucleus within the entire cell. A small value indicates a comparatively large nucleus compared to the cytoplasm.	None
Neighbours average volumes (neighbours_avg_volumes)	Accounts for the average volume of the surrounding cells.	$mm^3$
Neighbours average compactness (neighbours_avg_compactness)	Accounts for the average compactness of the surrounding cells.	None
Neighbours standard deviation of volumes (neighbours_std_volumes)	Measures the differences in sizes of the neighbouring cells.	$\mu m$
Neighbours standard deviation of compactness (neighbours_std_compactness)	Measures differences in compactness of the neighbouring cells.	None
Neighbours volume ratio (volume_neigh_ratio)	Ratio of the nucleus volume to the average volume of its neighbours.	None
Neighbours compactness ratio (compactness_neigh_ratio)	Ratio of the nucleus compactness to the average compactness of its neighbours.	None

#### Image level metrics

The cellular level metrics can be aggregated to create image level metrics:

- *Cell density* ($mm^{-2}$): total number of cells divided by the en-face area, which corresponds to skin surface.
- *Average volumes, compactness and distance to neighbours*: the overall averages for volumes, compactness and distances to neighbours are computed for the entire image.
- *Standard deviations for volumes and compactness*: the overall standard deviations for volumes and compactness are computed on the entire image.

### Cell level atypia definitions

To define atypia at the cellular level, we considered four approaches based on the quantitative metrics we obtained from deep learning segmentation (Table 2), extending previous qualitative studies on grading KC atypia with Reflectance Confocal Microscopy 2D images^14^. One simple, rule-based, expert definition is analysed while three Machine Learning based methods are also proposed.

All the approaches presented here share the same principle: atypia scores can be defined at cellular level thanks to the metrics derived from segmentations. Averaging these cell level atypia scores then gives a global atypia score to an entire 3D image. As we have the labels from histology for all the images, we can then compute a meaningful performance score at separating healthy versus pathological skins.

#### Rule based definition

Being able to segment and derive quantitative features at cellular level enable the use of rule-based definition for nucleus atypia. As an example of a simple rule, we define the following three criteria:

- Nuclei larger than 2.8 $\times\,10^{-7}$ µm are potential atypia (top 10% of the largest detected nuclei in our data).
- Nuclei with a compactness smaller than 0.592 are potential atypia (top 10% of the least compact detected nuclei in our data).
- Nuclei with neighbours larger than 1.56 $\times\,10^{-7}$ µm on average are potential atypias (top 10% of the largest detected neighbours in our data).

A cell will then be considered atypical if it meets at least two of these 3 criteria simultaneously.

#### Machine learning definitions

From the Machine Learning point of view, atypia detection can be seen as outlier detection^26^. The input features representing a nucleus are the feature derived from the segmentation and the output of the model is a binary score indicating whether a nucleus is atypical or not. Algorithms learn their own definition of atypia based on statistical evidence from the data. In this study, we consider two different approaches:

- An unsupervised approach with the Isolation Forest algorithm^27^, where atypia are simply considered as cells that look different from the majority of cells. No labels are needed for training the algorithm.
- A weakly-supervised approach, where the main assumption is that atypia mostly appear in pathological skin. During the training phase, all nuclei from pathological skin are considered atypical while all nuclei from healthy skin are considered healthy. Two different models are trained following this paradigm: a Logistic Regression^28^ and a XGBoost^29^.

Detailed explanations about the different algorithms, training procedures and outputs analysis can be found in the “Methods” section.

### Reader study

To compare the automated models to medical experts, we asked three dermatologists, highly experienced in non-invasive imaging, to review and assess an atypia score for each 3D image. The images are presented to the experts as two short videos, en-face and en-coupe views, spanning the entire 3D image.

Experts were asked to assess 3 criteria both on horizontal and vertical images (atypia related to the shape, size and spatial spread of atypia nuclei) using a scale from 0 to 4 (0 meaning no irregularities, 1 if less than 25%, 2 if between 25 and 50%, 3 between 50 and 75% and 4 if more than 75% of nuclei are irregular).

In total, 6 scores were given for each of the 185 LC-OCT 3D images of the study. For each score, a higher value indicates a higher degree of atypia. When summing all the scores, this gives a global atypia score ranging from 0 to 24. The score is normalized between 0 and 1 and compared to the Machine Learning based scores. A medical consensus score is computed by averaging the scores of each reviewer.

### Statistical tools

Classical statistical tools were used to analyse the different results of the study. To compare metrics between the healthy and pathological skins we used a T-test for the means of two independent samples of scores using scipy^30^. To compute the correlation between the scores of the different methods we used the Pearson correlation also using scipy.

## Results

### Statistically significant differences at image level

**Figure 2:**
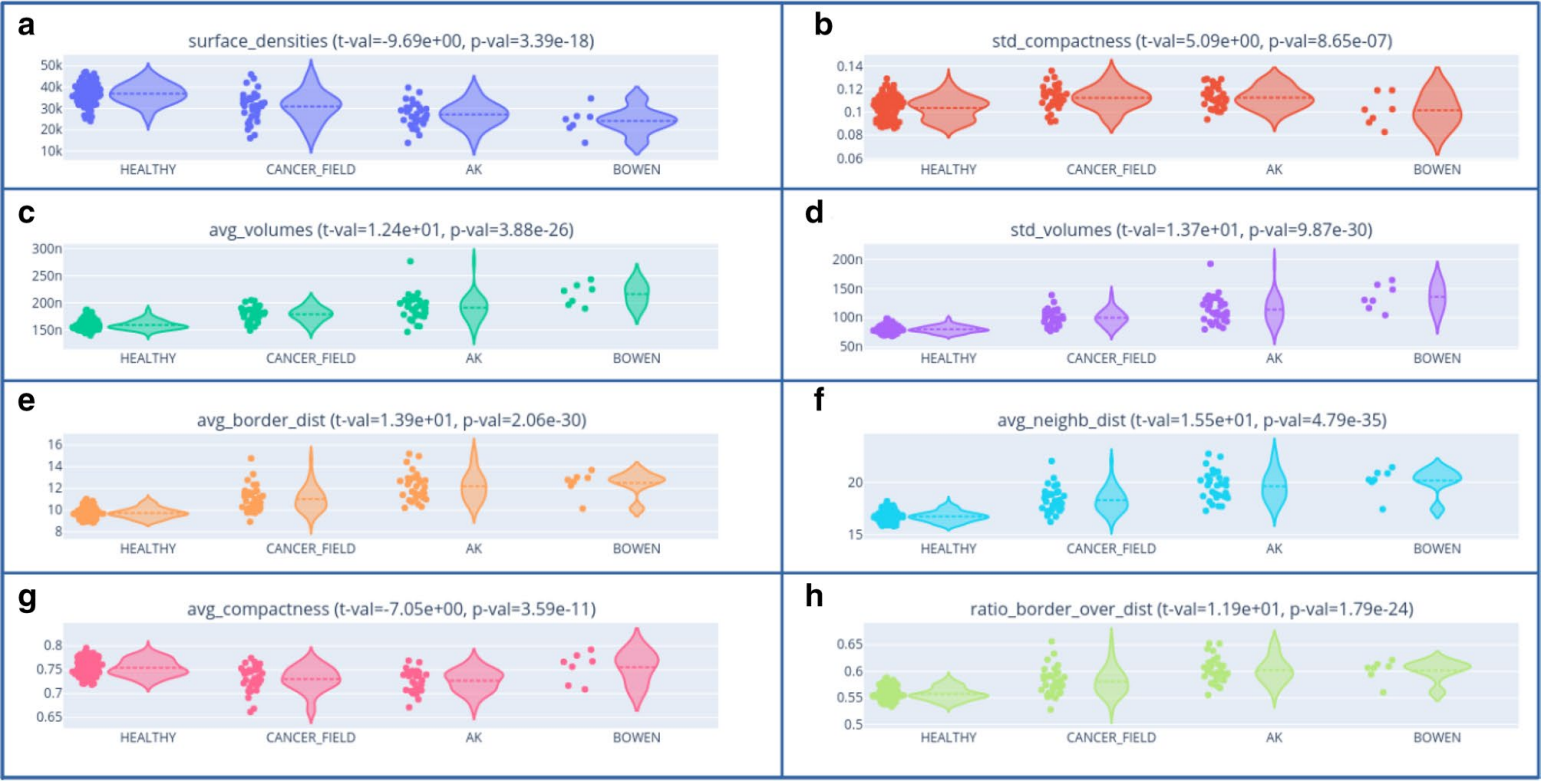
Global metrics per image with their corresponding t-values and p-values for the T-test for the means of two independent samples of scores.

A T-test analysis of the image level metrics shows statistically significant differences between healthy and pathological populations (Fig. 2). Positive t-values indicate larger values for the pathological population than the healthy population while negative value indicate the opposite.

Healthy skins have a higher cell density than pathological ones (Fig. 2a : t-value = 9.69, p-value = 3.39 $\times\,10^{-18}$) and larger nuclei compared to their cytoplasm as shown by a lower border to border distance over center to center distance ratio. (Fig. 2h: t-value = 11.9, p-value = 1.79 $\times\,10^{-24}$).

However pathological skins have larger nuclei in terms of volumes (Fig. 2c : t-value = 1.24, p-value = 3.88 $\times\,10^{-26}$). Moreover smaller and larger nuclei coexist in pathological skins while volumes are more uniform in healthy skins as shown by a higher standard deviation of volumes (Fig. 2d: t-value = 13.7, p-value = 9.87 $\times\,10^{-30}$). Pathological cells are also less compact (spherical) on average than healthy cells (Fig. 2g: t-value = 7.05, p-value = 3.59 $\times\,10^{-11}$) with more diversity in terms of compactness as shown by standard deviation of compactness (Fig. 2b: t-value = 5.09, p-value = 8.65 $\times\,10^{-7}$). Distances between nuclei from border to border (Fig. 2e: t-value = 13.9, p-value = 2.06 $\times\,10^{-30}$) and from center to center (Fig. 2f: t-value = 15.5, p-value = 4.79 $\times\,10^{-35}$) are greater for pathological skins.

**Table 3:** AUC scores for different subsets of the data for different methods (and their Pearson correlation to XGBoost and p-values).

	XGBoost	Simple Rule	Logistic Regression	Isolation Forest	Medical Consensus	Expert 1	Expert 2	Expert 3
Healthy vs pathological	0.982	0.965 (0.92, p = 4e $\times\,10^{-78}$)	0.970 (0.98, p = 1.7 $\times\,10^{-128}$)	0.971 (0.88, p = 3.7 $\times\,10^{-62}$)	0.766 (0.66, p = 4 $\times\,10^{-24}$)	0.715 (0.45, p = 9 $\times\,10^{-11}$)	0.745 (0.54, p = 2 $\times\,10^{-15}$)	0.708 (0.47, p = 8 $\times\,10^{-12}$)
Healthy vs AK and Bowen	0.991	0.968 (0.92, p = 3 $\times\,10^{-67}$)	0.979 (0.98, p = 9.9 $\times\,10^{-112}$)	0.978 (0.89, p = 9.5 $\times\,10^{-54}$)	0.952 (0.75, p = 1 $\times\,10^{-29}$)	0.828 (0.52, p = 4 $\times\,10^{-12}$)	0.924 (0.63, p = 5 $\times\,10^{-18}$)	0.839 (0.53, p = 2 $\times\,10^{-12}$)
Healthy vs subclinical AK	0.972	0.962 (0.84, p = 1 $\times\,10^{-40}$)	0.960 (0.96, p = 8 $\times\,10^{-84}$)	0.964 (0.78, p = 2 $\times\,10^{-31}$)	0.563 (0.27, p = 6 $\times\,10^{-4}$)	0.592 (0.12, p = 1 $\times\,10^{-1}$)	0.550 (0.21, p = 1 $\times\,10^{-2}$)	0.564 (0.18, p = 1 $\times\,10^{-2}$)

### Discrimintative power of automated cell level atypia definitions

By averaging the individual nuclei scores by 3D images for each method, we can compare the discriminative powers of the rule-based, the unsupervised and the weakly-supervised models to differentiate between healthy skin on one hand and pathological skin (AK, SAK or Bowen) on the other. These scores are computed at image level and compared to histopathology groundtruth results. As shown in Fig. 4, all Machine Learning approaches show a better discriminative power than the rule-based approach (AUC = 0.965), with an AUC of 0.970 for the Logistic Regression, 0.971 for the Isolation Forest and 0.982 for XGBoost. Overall, the weakly-supervised models are outperforming the unsupervised one.

**Figure 3:**
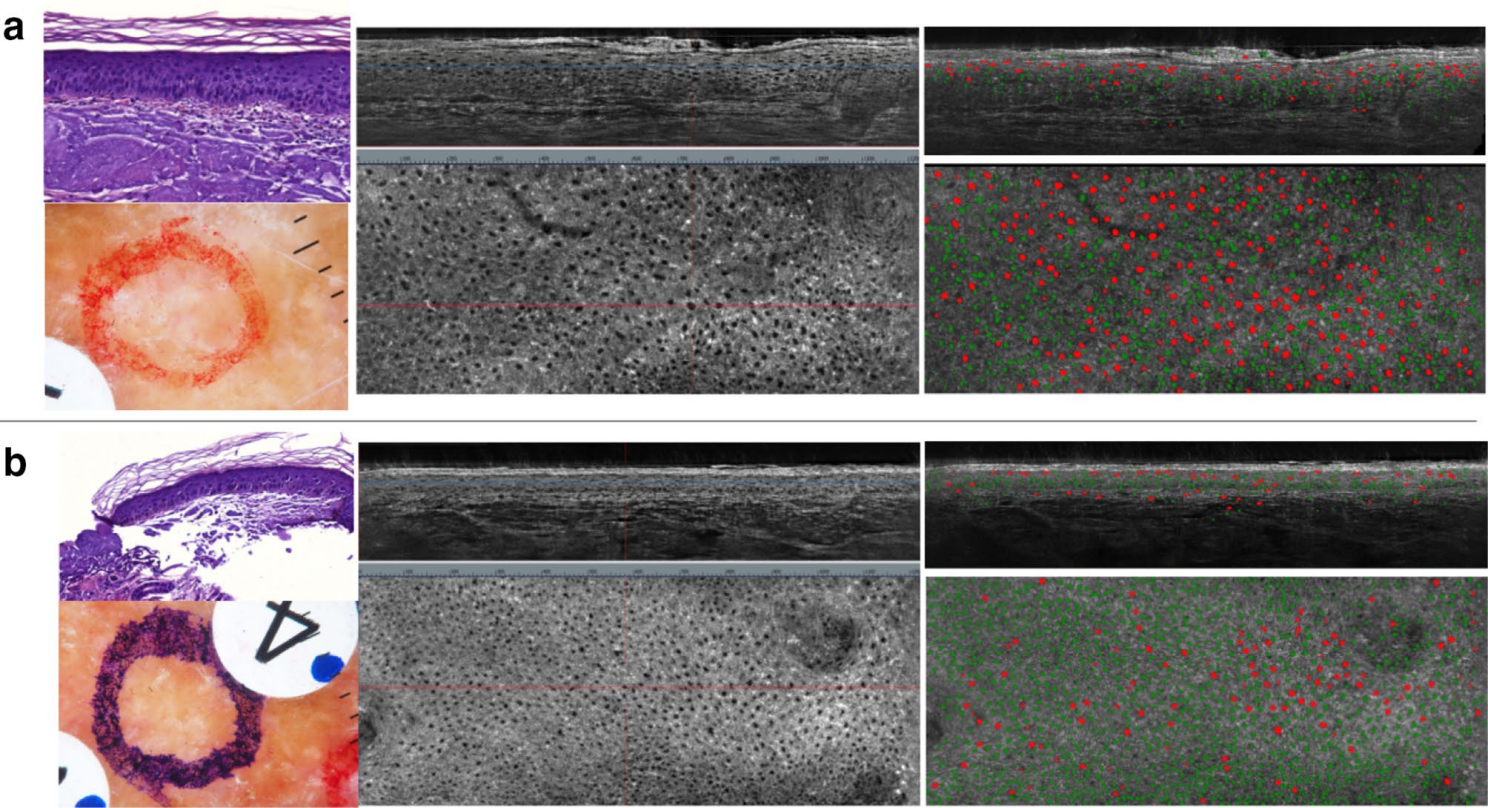
Example of atypia detections (in red) and normal nulcei (in green) with the simple rule-based atypia definition for one AK lesion (**a**) and its perilesional field of cancerization (**b**).

**Figure 4:**
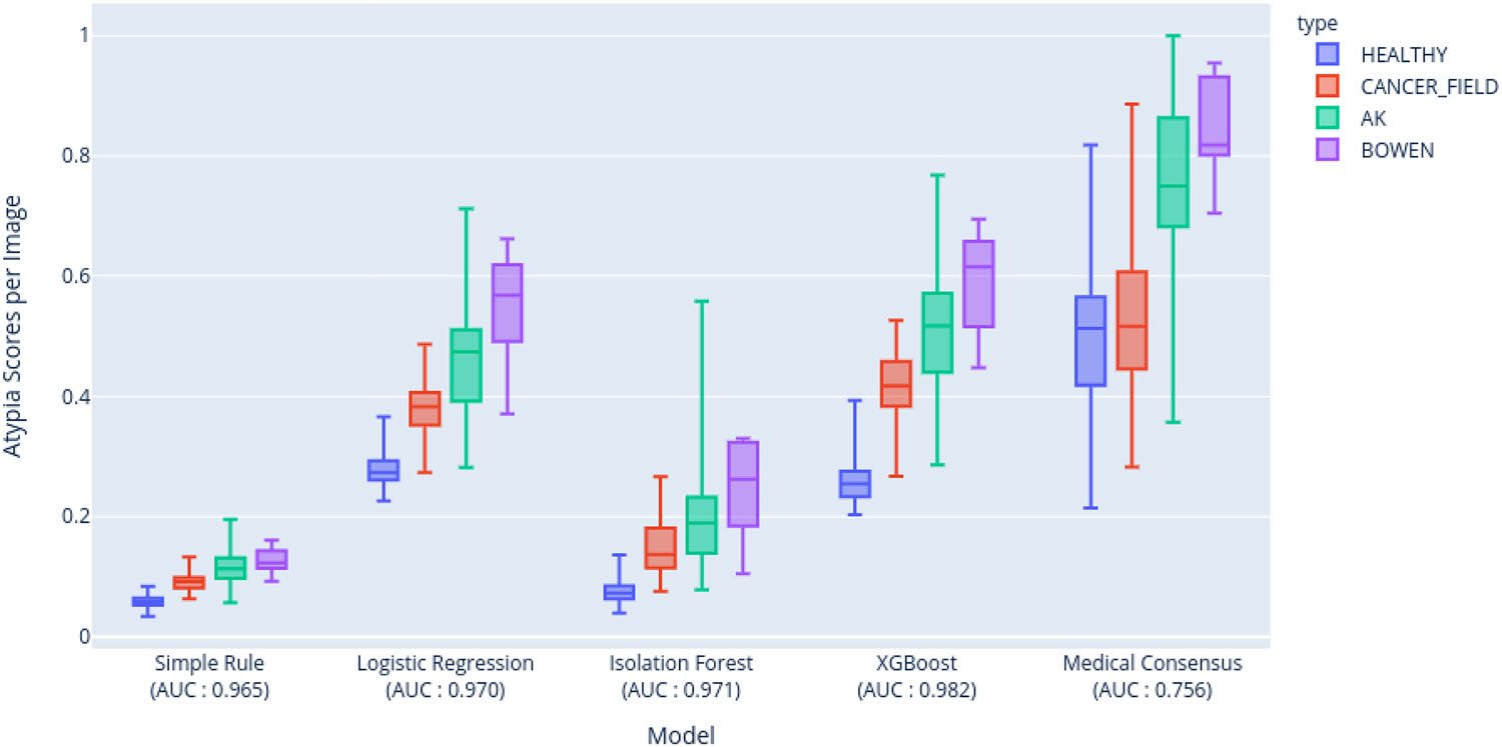
Box plots (min, max, median, q1, q3) of average atypia score per stack for different methods including medical consensus from the reader study. The medical consensus has a much lower AUC score than the other automated methods.

### Automated methods outperform medical experts assessing an atypia score

The medical consensus of experts from the reader study achieves an AUC score of 0.766 for the entire dataset (while individual expert have scores ranging from 0.708 to 0.745), under-performing automated scores by more than 20 points of AUC (Fig. 4). Nevertheless statistically significant positive Pearson correlations of 0.647 (p = 2.7 $\times\,10^{-23}$), 0.592 (p = 6.7 $\times\,10^{-19}$) and 0.657 (p = 4.1 $\times\,10^{-24}$) can be observed with the Logistic Regression, the Isolation Forest and XGBoost respectively. In more details, as shown on Table 3, medical consensus is good at differentiating between healthy skin and AK or Bowen, where there is a clear-cut visible difference of atypia within images, with an AUC of 0.952, which is only slightly worse than the automated approaches (ranging from 0.968 to 0.991). However, medical consensus can hardly discriminate between healthy skin and field of cancerization with an AUC of 0.573. Looking at each of the 6 different scores individually yields similar results.

## Discussion

In this study, we demonstrated the ability to apply deep learning models to segment nuclei on LC-OCT 3D images which is an impossible task for humans in a reasonable amount of time. The inference time of around 3 minutes of the deep learning model for a fully automated procedure could allow physician to benefit from all the results presented in this study during their daily practice.

The metrics derived from the segmentations at image level showed statistical differences which are intuitive to clinical experts : pathological skins often present larger, less spherical nuclei, with more heterogeneity. The ability to precisely measure those intuitive metrics is an important step towards objective measurements and standardized clinical decisions.

The cohort used for this study is of a moderate size but 185 3D images are sufficient to obtain significant results to compute and compare discriminative powers of the different atypia scoring methods. Even though the two populations do suffer some biases, the hypothesis validated by medical experts was that the differences between lesional and non-lesional epidermis exceed by far the variability of epidermis that may be attributed to the age, gender and localization differences in between our two cohorts. Moreover, the automated pipeline used in this study only uses the raw images without taking into account any of these parameters.

Defining what cellular atypia or dysplasia means is a difficult task since these concepts rely on the expertise of pathologists and not on quantitative metrics^31^. Nevertheless, we showed that a simple rule-based definition of atypia can quantify atypia with a better precision than medical experts.

With a similar rule-based approach, more complex rules could be defined by experts in order to get definitions of atypia with better discriminative powers. Moreover, it would be possible, as a direct extension of this work, to apply several rule-based classifications derived from expert knowledge to distinguish between different kinds of atypia. These definitions apply at cell level and can be used to highlight atypia during a clinical review as shown in Fig. 3.

**Figure 5:**
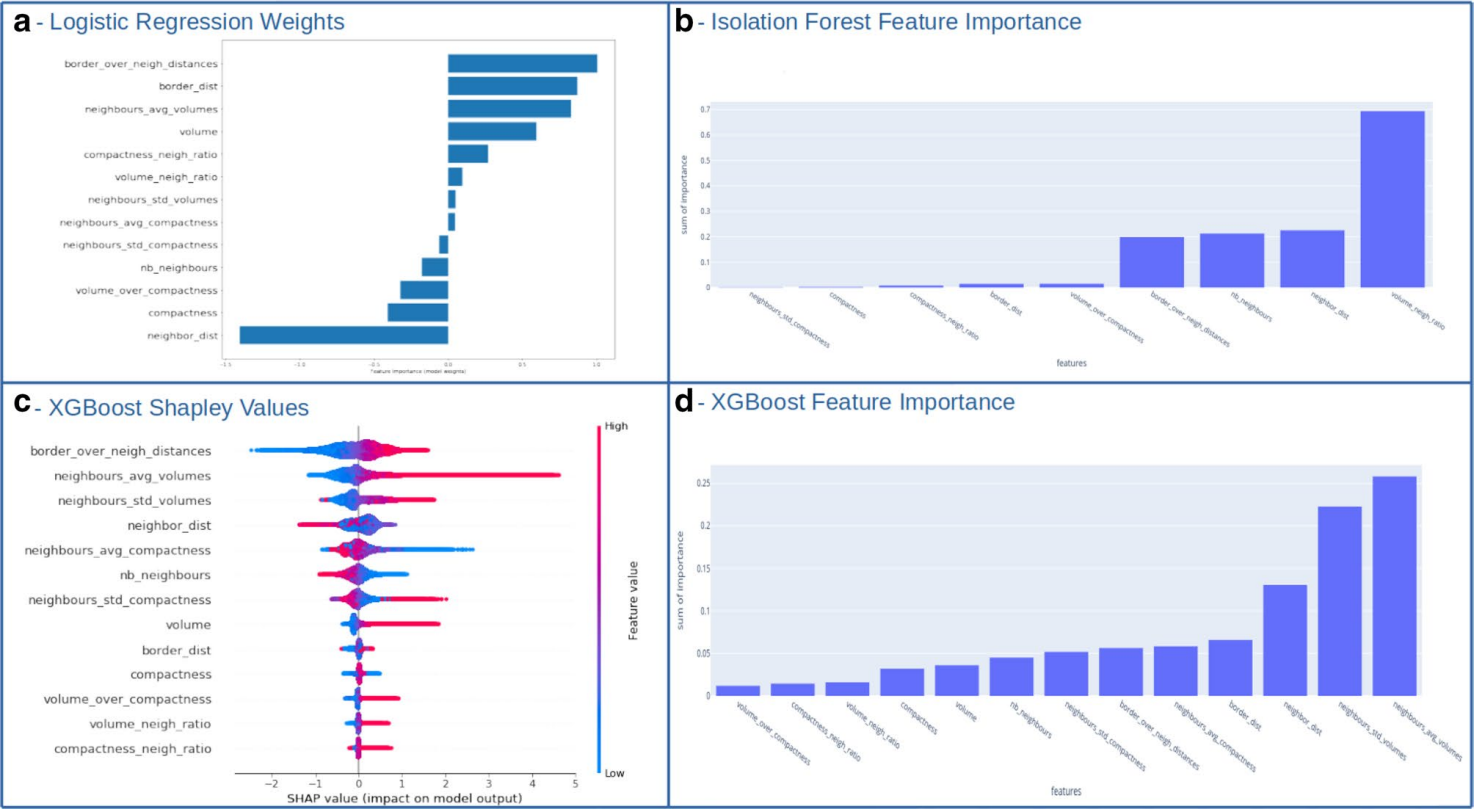
Models atypia definition interpretability: (**a**) Logistic regression model weights (**b**) Isolation Forest global feature importance (**c**) XGBoost Shapley values (**d**) XGBoost global feature importance.

Nevertheless, expert rules are not complex enough to model the interactions between 13 features and may result in an overly simplified definition of atypia. That is why data-driven definitions of atypia based on Machine Learning show better performances than rule-based methods. The more complex the model the harder it is to interpret, and there is always a trade-off between interpretability and performance. However, it is possible to analyse the feature importance for each model as shown in Fig. 5 to give more trust to the practitioners in the automated scores.

For the Logistic Regression, the perfect prototype for an atypical cell is a large (both large nucleus volume and cytoplasm radius), non spherical (low compactness) cell, surrounded by a few large cells as neighbours. For the XGBoost algorithm, the perfect prototype for an atypical cell is also large cell surrounded by a few heterogeneous cells in terms of sizes as shown in Fig. 5c,d. For the Isolation Forest 4 features are mostly used to define atypia (Fig. 5b): the volume ratio with neighbouring cells, the distance and number of neighbouring cells and the cytoplasm size.

Even though the definitions of atypia are different for each model they are highly correlated between each other: the Logistic Regression has a Pearson correlation coefficient at the global atypia level of 0.98 and 0.95 with XGBoost and the simple rule respectively while the Isolation Forest has a correlation of 0.93, 0.88 and 0.91 with the simple rule, the Logistic Regression and XGBoost respectively. These correlations suggest that the most complex and best performing algorithm can be safely used without losing the general understanding of what atypia intuitively means. Moreover it is remarkable that for the four automated approaches, we see that (Fig. 4) the ranking between subclinical AK, AK and Bowen is aligned with the expected clinical output with growing scores of atypia, while no distinction was made between them during training.

The medical consensus defined in the reader study, only created from the scoring of three experts, is not designed to accurately quantify the capacity of human experts on this task. It is rather meant to give an overview of how hard it is for the human eyes to grasp information from so many nuclei in 3D and assign to it a global score of atypia. We can see from Table 3 that the medical consensus reaches a decent score when comparing clear-cut lesions: healthy skin versus AK and Bowen. The consensus has a statistically significant correlation with XGBoost scores of 0.66. However, the performances and level of correlation to XGBoost drops when comparing healthy skin versus subclinical AK which is a harder task. The same observation as for the consensus results can be made for each expert individually but averaging the scores to create medical consensus significantly improves the results compared to individual contributions, slightly lowering the gap between automated approach and individual expert scores. These results show that subtle differences are really difficult to be accurately quantified by human experts while automated methods still perform at a very high level. Since detecting cancers as early as possible is beneficial for treatment and survival chances, it’s particularly important to improve on non clear-cut lesions. The fact that our methods outperform individual experts and the medical consensus show that a precise automated atypia score would be a precious piece of information for clinicians to assist them in making their diagnoses in the best interest of their patients.

We believe that this technology, able to obtain a reproducible atypia score, robust to subtle changes, in a few minutes and non-invasively brings a response to two of the currently unmet needs for AK treatments according to the International Dermatology Outcome Measures^32^: the measurement of AK in clinical practice and the outcomes of clinical trials. It opens a wide range of promising applications. For the dermatologists it will add a powerful assistance to quantify the level of atypia of a lesion but also to precisely monitor the evolution of a lesion overtime. For medical research on cancer treatment, it will allow quantitative analysis of treatment efficiency. A standardized metric can also help compare results of different treatments between each other.

Further research should focus on applying similar approach to different pathologies. In particular, targeting melanocytes instead of keratinocytes alongside with designing problem specific features (e.g. using the pixel intensity to characterize melanin) is a promising path for the assisted diagnosis of melanoma skin cancers. Another path we wish to follow is the use of our work to demonstrate the efficiency of a cancer treatment in a clinical trial.

## Methods

The study was approved by the ethical committee of the university hospital of Saint-Etienne (NCT0.3731247) and was conducted in accordance with the ethical principles of the Helsinki Declaration.

### Deep Learning: StarDist training procedure

A 3D image of in-vivo skin with a field of view of 0.4mm × 0.5mm × 1.2mm can contain tens of thousands of keratinocytes inside the epidermis. Annotating them in 3D is a very tedious and complicated task but is absolutely mandatory in order to train a deep learning algorithm.

A semi-automatic software developed by DAMAE Medical allowed human experts to annotate 50 healthy LC-OCT 3D images coming from different volunteers, on different body sites and 20 pathological skins so that atypical nuclei are available to the algorithm during training. All the training images are completely disjoint of the images of this study.

This amount of data might not seem huge, but there are multiple reasons why the training of a deep learning algorithm is feasible here:

- Annotated 3D images are much larger than the training patch size (60 × 128 × 128): a 3D images of size 300 × 400 × 1200 contains approximately 150 non-overlapping 60 × 128 × 128 patches. This represents more than ten thousand different training patches.
- One training patch contains hundreds of nuclei, so overlapping patches are still very different images, making the actual × 150 cropping boost much bigger.
- Data augmentations (randoms flips, intensity changes, crops and zooms) prevent overfitting and allow the model to learn essential features to detect nuclei.

We used the open source library [https://github.com/stardist/stardist] of the original StarDist^19,33^ paper to train our own 3D StarDist model for LC-OCT 3D images. The model is trained for 200 epochs with a batch size of 2 and an exponential learning rate decay of 0.8 every 10 epochs, the best iteration on the validation set (20% of the stacks) is kept for the final model. The Non-Maximum-Suppression (NMS) algorithm applied at post-processing enforces the detection of non-overlapping cells. In order to allow adjacent cells to be detected, the NMS threshold was set to 0.05 (meaning that two detected cells can’t overlap on more than 5% of their volumes). Each detected nucleus is represented by a central position and 96 rays of different length providing an accurate 3D representation of the nucleus with a large variety of possible shapes.

### Stratified 5-fold cross validation

For all the experiments where a Machine Learning algorithm was trained to score atypia, 5-fold stratified cross-validation was performed at the image level. At first, the 185 LC-OCT 3D images are separated into 5 folds, each of them containing a similar proportion of healthy and pathological images, then a model is trained using nuclei coming from 4 folds and validated on completely unseen images belonging to the last fold. This represents a total of more than 3.7 million cells to be classified as normal or atypical. This approach allows to observe reliable results across the entire database limiting the risks of over-fitting compared to using a predetermined subset used as hold-out validation^34^.

### Unsupervised approach

The unsupervised approach uses no labels and no prior assumption about cellular atypia. The idea is to analyze all the detected nuclei from both healthy and pathological skins and derive a mathematical frontier separating ’normal’ nuclei from ’outliers’ (or atypia).

We used the Isolation Forest algorithm to see if nuclei isolated by the algorithm were predominant in pathological 3D images. Even though this approach does not require any label, the 5 fold cross-validation scheme was used to allow a fair comparison with the weakly-supervised learning approach. We used scikit-learn’s^35^ implementation with default parameters.

Without supervision, the notion of outlier learnt by the model could be completely different from cellular atypia. However the high discriminative power (AUC = 0.971 as shown in Table 3) of the aggregated results at the image level gives a good guarantee that outliers detected by the model are indeed atypical cells.

In order to better understand what is considered as atypical for the model we derived the global feature importance of the model^36^. Figure 5b shows that the 4 most important features are related to neighbouring cells, giving interesting hints of its definition of atypia.

However, the Isolation Forest is a quite complex Machine Learning model (based on a Random Forest and the number of splits required to predict an instance), so its definition of atypia cannot be easily summed up in one sentence as feature interactions play an important role in the model decisions. Using a simpler model in a supervised setting is a good way to overcome this issue as discussed below.

### Weakly-supervised approach

A fully supervised approach for this study would need one label per detected cell, this means more than 3.7 million labels done by human experts, which is infeasible. Moreover, no perfect ground truth could be achieved since the visual definition of atypia remains blurry. The weakly-supervised approach hence only uses a simple prior assumption: atypia must be significantly more frequent within pathological skin than healthy skin.

In this setting, nuclei from pathological images are all considered as atypia while nuclei from healthy images are considered healthy. Even though this might seem a very naive proxy for atypia, this kind of weakly-supervised approach have already been successfully used for detection of visual anomalies in histology images^37^. Since we have the clinical diagnoses for each image, we can use them to create weak labels.

#### Logistic regression

In order to be able to easily understand the final model of the weakly-supervised approach we trained a simple Machine Learning model: a logistic regression^28^. Logistic Regression is probably the simplest yet powerful algorithm for binary classification. It has the advantage of being easily comprehensible thanks to its closed form prediction function

$$\begin{aligned} p= \sigma (\beta _0 + \beta _1 x_1 + \dots + \beta _n x_n) \end{aligned}$$

with *p* being the probability of being an atypia, $x_i$ the input features, $\sigma$ the sigmoid function and $\beta _i$ the learnt parameters. Before training, features are normalized to get 0-mean and standard deviation of 1. This is done following the safe cross validation procedure in order to get reliable results : standardization is done with training statistics and the validation set is transformed accordingly. As we have weak labels, the default scikit-learn’s^35^ parameters are used without need for hyper-parameter tuning nor risks of overfitting.

Using 5 fold cross-validation at image level, we can predict for each nucleus an atypia score between 0 and 1. Having a continuous probability as atypia score allows to set up a binary threshold to detect atypia. The choice of this threshold could allow us to sharpen our definition of atypia, for example a threshold of 0.5 will consider more nuclei as atypical when a threshold of 0.9 will only detect the most atypical nuclei. The results shown in Fig. 4 are obtained using a simple averaging of all cells predictions, without thresholding.

Moreover, we can use the fitted parameters of the logistic regression to understand what atypia means for the trained model. Figure 5a shows the model weights: the larger the absolute value the more impact the feature has on the final decision. Positive weights mean positively correlated impact (a larger value of the feature means a larger atypia score) while negative weights mean the opposite. We can understand in depth how atypia is defined by the model. Cytoplasm ratio (border to border distance divided by center to center distance), nucleus volume and neighbours volumes are positively correlated to atypia while neighbours distance, number of neighbours and volume over compactness are negatively correlated to atypia.

#### XGBoost

XGBoost^29^ is based on gradient boosted trees method and is one the best algorithm to solve tabular data tasks. It has a much bigger modeling capacity than a logistic regression and heavily relies on feature interactions. It outperformed all other approaches for this study with an AUC score of 0.982 . Because of the weak labels paradigm, no tuning has been performed and a simple model with 200 estimators (no early stopping), a depth of 5 and sub sample rate of 0.5 was used.

Despite the complexity of the model, it is interpretable thanks to shapley values^38^ as shown in Fig. 5c. This graph allows a similar interpretation as a logistic regression, although more sophisticated. We can see that cytoplasm ratio, volumes and standard deviations of neighbours are positively correlated to atypia score while distance to neighbours, compactness and number of neighbours are negatively correlated to atypia score.
